# Sulforaphane suppresses lipopolysaccharide‐ and Pam3CysSerLys4‐mediated inflammation in chronic obstructive pulmonary disease via toll‐like receptors

**DOI:** 10.1002/2211-5463.13118

**Published:** 2021-05-01

**Authors:** Xiaoli Zeng, Xiaoju Liu, Hairong Bao

**Affiliations:** ^1^ Department of Gerontal Respiratory Medicine The First Hospital of Lanzhou University China

**Keywords:** chronic obstructive pulmonary disease, inflammation, myeloid differentiation factor 88, sulforaphane, toll‐like receptors

## Abstract

Chronic obstructive pulmonary disease (COPD) is a progressive inflammatory disease of the airway that represents a large global disease burden. Inflammation is a prominent feature of COPD and represents an important target for treatment. Toll‐like receptors (TLRs) are pattern recognition receptors that detect invading microorganisms and nonmicrobial endogenous molecules to trigger inflammatory responses during host defense and tissue repair. The TLR signaling pathway is closely linked to the pathogenesis of COPD. Sulforaphane (SFN), an isothiocyanate derived from cruciferous vegetables, is well known for its anti‐inflammatory activities. However, the molecular function of SFN in inhibition of COPD inflammation has yet to be fully elucidated. In this study, we investigated the effects of SFN on lipopolysaccharide (LPS)‐ or Pam3CysSerLys4 (Pam3CSK4)‐induced inflammation in monocyte‐derived macrophages (MDMs) from patients with COPD. MDMs from patients with COPD showed higher expression levels of TLR2, TLR4 and downstream myeloid differentiation factor 88 (MyD88) than healthy controls, along with increased secretion of interleukin‐6 (IL‐6) and tumor necrosis factor‐α (TNF‐α) (*P* < 0.05). Stimulation with TLR ligands (Pam3CSK4 and LPS) up‐regulated the levels of TLR2, TLR4 and MyD88 in MDMs from patients with COPD and induced the release of IL‐6 and TNF‐α (*P* < 0.05). Pretreatment of MDMs from patients with COPD with SFN significantly suppressed Pam3CSK4‐ or LPS‐induced TLR2, TLR4 and MyD88 expression, along with a reduction in the production of IL‐6 and TNF‐α (*P* < 0.05). Collectively, these data indicate that SFN exerts its anti‐inflammatory activity in COPD by modulating the TLR pathway. SFN may represent a potential therapeutic agent for the treatment of COPD.

## Graphical abstract

Chronic obstructive pulmonary disease (COPD) is a chronic inflammatory disease of the airway, and toll‐like receptors play important roles in the regulation of inflammatory processes. In this study, we report that sulforaphane exerts anti‐inflammatory activity via the toll‐like receptor pathway in macrophages from patients with COPD. Sulforaphane may have potential as a new therapeutic approach for the anti‐inflammatory treatment of COPD.

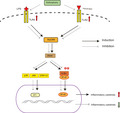

**Abbreviations**
AM: alveolar macrophage
COPD: chronic obstructive pulmonary disease
FEV_1_: forced expiratory volume in 1 s
GM‐CSF: granulocyte‐macrophage colony‐stimulating factor
IL: interleukin
LPS: lipopolysaccharide
MDM: monocyte‐derived macrophage
MyD88: myeloid differentiation factor 88
NF‐κB: nuclear factor‐κB
Nrf2: nuclear factor erythroid 2 related factor 2
Pam3CSK4: Pam3CysSerLys4
PAMP: pathogen‐associated molecular pattern
SD: standard deviation
SFN: sulforaphane
TLR: toll‐like receptor
TNF‐α: tumor necrosis factor‐α

Chronic obstructive pulmonary disease (COPD) is a common respiratory disease that is characterized by chronic inflammation of the lungs in response to noxious particles or gases, primarily smoking [1]. COPD is one of the leading causes of disability, morbidity and mortality worldwide. At present, there are approximately 210 million people suffering from some degree of COPD. This disease causes at least 3 million deaths each year, corresponding to 5% total global mortality [2]. Current treatments involving the combination of bronchodilators and corticosteroids relieve symptoms only temporarily but cannot delay or cure the disease [1]. Therefore, it is imperative to identify other effective therapeutic targets and strategies.

Inflammation is a critical feature in the development of COPD. Inflammatory cells and mediators have been implicated in the progression of COPD [3]. Of these inflammatory cells, macrophages play an important role. Cigarette smoke and noxious particles can stimulate macrophages in the airways. Once activated, these macrophages release inflammatory mediators and chemokines, including interleukin (IL)‐6, IL‐8, tumor necrosis factor‐α (TNF‐α), monocyte chemotactic protein‐1 and proteolytic enzymes; these mediators contribute to the formation of pulmonary emphysema [4].

Toll‐like receptors (TLRs) are a class of conservative pattern recognition receptors that play important roles in the regulation of immune and inflammatory processes. TLR2 and TLR4 have been regarded as the main sensors for recognizing pathogen‐associated molecular patterns (PAMPs) from Gram‐positive and Gram‐negative bacteria, respectively [5]. After ligand binding, both TLR2 and TLR4 function in the myeloid differentiation factor 88 (MyD88)‐dependent pathway [6]. MyD88 is the central adaptor protein for signal transduction of TLRs and interacts with TLRs, leading to activation of the mitogen‐activated protein kinase cascade and nuclear factor‐κB (NF‐κB), causing expression of proinflammatory mediators [7]. Several studies have confirmed that a significant proportion of patients with COPD exhibit bacterial colonization in the lower airway involving a wide spectrum of pathogens, including *Streptococcus pneumoniae*, *Moraxella catarrhalis* and *Haemophilus influenzae* [8, 9]. The bacterial PAMPs, lipopolysaccharide (LPS) and lipopeptide, are transduced by TLR4 and TLR2, respectively, thus promoting the secretion of proinflammatory mediators.

Sulforaphane (SFN) is a naturally occurring isothiocyanate obtained from cruciferous vegetables, such as broccoli or cabbages, that exhibits anti‐inflammatory, antioxidation and anticancer properties [10]. SFN can reduce inflammatory biomarkers in multiple signaling pathways and provide probable protection for human health against chronic diseases [11]. Harvey *et al*. [12] showed that SFN treatment restored bacteria recognition and phagocytosis in alveolar macrophages (AMs) from patients with COPD through activating nuclear factor erythroid 2 related factor 2 (Nrf2), to prevent exacerbation of COPD caused by bacterial infection. Starrett and Blake [13] demonstrated that SFN inhibited cigarette smoke extract‐induced IL‐8 and monocyte chemotactic protein‐1 production in human epithelial cells. Our previous study found that SFN could inhibit the expressions of TLR4 and MyD88 and exert an anti‐inflammatory effect in COPD [14]. However, the contribution of the TLR2/MyD88 pathway to the inflammation, how TLR2 and TLR4 simultaneously affect inflammation, and the effect of SFN on it remain to be studied. Therefore, in this study, we investigated the effect of SFN on inflammation in COPD and attempted to provide assistance in finding new drugs with fewer side effects.

## Materials and methods

### Subjects

This study was approved by the ethics committee of the First Hospital of Lanzhou University and was carried out in accordance with the Declaration of Helsinki. All subjects provided written informed consent. We recruited 25 patients diagnosed with COPD at the Department of Gerontal Respiratory Medicine, The First Hospital of Lanzhou University. COPD diagnosis was based on the 2017 global initiative for chronic obstructive lung disease (GOLD) Guidelines [1]; forced expiratory volume in 1 s (FEV_1_)/forced vital capacity of every patient with COPD was lower than 70% (the standard set by the GOLD Guidelines). We also recruited 25 healthy control subjects with normal spirometry. The characteristics of our subjects are given in Table 1. Subjects with a history of asthma, other pulmonary diseases or allergic diseases were excluded from the study.

**Table 1:** Characteristics of the study subjects. F, female; FVC, forced vital capacity; ICS, inhaled corticosteroid; LABA, long‐acting beta 2‐agonist; LAMA, long‐acting muscarinic antagonist; M, male.

	Healthy control subjects	Patients with COPD
Subjects (*n*)	25	25
Age (years) (range)	68 ± 2 (53–76)	70 ± 8 (53–89)
Sex (M/F)	20/5	16/9
Smoker (former/never)	14/11	15/10
Smoking (pack‐years)	30 ± 14	34 ± 19
Inhaled medication (*n*)		25
LAMA		15
LAMA+LABA		6
LAMA+LABA+ICS		4
FEV_1_ % predicted	89 ± 10	50 ± 17^a^
FEV_1_/FVC	87 ± 7	53 ± 12^a^

^a^ *P* < 0.01 versus healthy control subjects. Data were compared by two‐tailed Student's *t*‐test and chi‐square test.

### Preparation of monocyte‐derived macrophages

Blood samples (20 mL) were collected from patients with COPD and healthy control subjects by antecubital venipuncture into heparinized tubes. Peripheral blood was then diluted with PBS (1 : 1), loaded on Ficoll–Hypaque gradient (Solarbio, Beijing, China) and centrifuged for 400 ***g*** for 15 min at room temperature. Peripheral blood mononuclear cells were then collected and resuspended in complete RPMI 1640 medium (Gibco, Grand Island, NY, USA) containing 10% fetal calf serum (HyClone, GE Healthcare, Chicago, IL, USA), 2 mm l‐glutamine, 100 IU·mol^−1^ penicillin and 100 IU·mol^−1^ streptomycin. Next, cells were seeded into 24‐well plates (2.0–2.5 × 10^6^ cells/well). After 2 h, nonadherent cells were removed, and adherent monocytes were cultured in the presence of 2 ng·mL^−1^ granulocyte‐macrophage colony‐stimulating factor (GM‐CSF; R&D Systems, Minneapolis, MN, USA) at 37 °C in a 5% CO_2_ incubator for 12 days to generate monocyte‐derived macrophages (MDMs) as Taylor *et al*. [15] described.

### Cell treatments with Pam3CysSerLys4, LPS or SFN

MDMs were treated with a TLR4 agonist (LPS; Sigma‐Aldrich, St. Louis, MO, USA) or TLR2 agonist (Pam3CysSerLys4 [Pam3CSK4]; InvivoGen, Carlsbad, CA, USA) and/or SFN (Sigma‐Aldrich). To investigate the optimal intervention concentrations, we treated MDMs with 1, 10, 100 and 1000 ng·mL^−1^ LPS; 1, 10, 100 and 1000 ng·mL^−1^ Pam3CSK4; or 2.5, 5, 10 and 20 μmol·L^−1^ SFN for 6 h. Subsequently, cells were harvested and used for quantitative real‐time PCR. According to the PCR results, we then selected Pam3CSK4 (1000 ng·mL^−1^), LPS (1000 ng·mL^−1^) and SFN (20 μmol·L^−1^) for all subsequent experiments. To evaluate the effects of SFN on the TLR/MyD88 pathway, we pretreated MDMs with SFN (20 μmol·L^−1^) for 1 h and then challenged these cells with Pam3CSK4 or LPS (1000 ng·mL^−1^). After 6 h of incubation, the cells were harvested for gene analysis; protein measurements were taken at 24 h.

### Quantitative real‐time PCR assay

Total RNA was extracted from MDMs using the RNAiso Plus reagent (Takara, Tokyo, Japan) in accordance with the manufacturer's instructions. Isolated total RNA was then reverse transcribed into cDNA using PrimeScript™ Reverse Transcriptase (Takara). Quantitative real‐time PCR was performed on a Rotor‐gene 6000 Real‐Time Thermal Cycler (Corbett, Sydney, NSW, Australia), using SYBR^®^ Premix Ex Taq™ II kit (Takara). The PCR cycle was as follows: 95 °C for 30 s, 95 °C for 5 s and 60 °C for 30 s; these conditions were repeated for 40 cycles. The expression levels of genes were then calculated according to the Livak method ($2^{‐\Delta \Delta C_{T}}$) and normalized to the GAPDH housekeeping gene. Primer sequences are as follows: TLR2: 5′‐CAGG AGCTCTTAGTGACCAAGTGAA‐3′ (forward), 5′‐CACAAAGTATGTGGCATTG TCCAG‐3′ (reverse), TLR4: 5′‐AGGATGATGCCAGGATGATGTC‐3′ (forward), 5′‐TCAGGTCCAGGTTCTTGGTTGAG‐3′ (reverse), MyD88: 5′‐AGCCAGGCTG GAGCAAGGTA‐3′ (forward), 5′‐GGCAGCTAAATGCCTCAACAAGA‐3′ (reverse), GAPDH: 5′‐GCACCGTCAAGGCTGAGAAC‐3′ (forward), and 5′‐TGGTGA AGACGCCAGTGGA‐3′ (reverse).

### Western blotting analysis

The radioimmunoprecipitation assay method was used to extract total protein from cells. Protein samples (40 μg) were separated by 10% SDS‐PAGE and transferred to polyvinylidene fluoride membrane using a wet transfer cell (Bio‐Rad, Hercules, CA, USA). Blots were blocked with Tris‐buffered saline Tween buffer containing 5% skimmed milk for 60 min at room temperature and then incubated overnight with primary antibodies, including anti‐TLR2, anti‐TLR4, anti‐MyD88 and anti‐β‐actin IgG (Santa Cruz, Inc., Dallas, TX, USA) at 4 °C. After washing with Tris‐buffered saline Tween buffer, the blots were incubated with a secondary antibody (goat anti‐mouse IgG or goat anti‐rabbit IgG) for 2 h at room temperature. The membranes were then rewashed, and antibody binding was detected by an enhanced chemiluminescence kit. β‐Actin expression was used as an internal control to demonstrate equal loading of protein samples.

### Cytokine measurements

Twenty‐four hours after the stimulation of TLRs, we measured the concentrations of IL‐6 and TNF‐α in cell supernatants using ELISA kits (R&D Systems); these kits were used in accordance with the manufacturer's instructions.

### Statistical analysis

spss 19.0 software (SPSS Inc., Chicago, IL, USA) was used for all statistical analyses. Data were expressed as means ± standard deviation (SD). First, homogeneity of variance was analyzed by Levene's test, then Student's *t*‐test was used for comparison between two groups, and one‐way ANOVAs for comparison between more than two groups were performed (*P* > 0.05 by Levene's test). Categorical data were analyzed by the chi‐square test, and the correlation coefficients were obtained by Pearson test. A *P* value <0.05 was accepted as statistically significant.

## Results

### TLRs agonists and SFN affected the expression of TLRs in a dose‐dependent manner

**Fig. 1:**
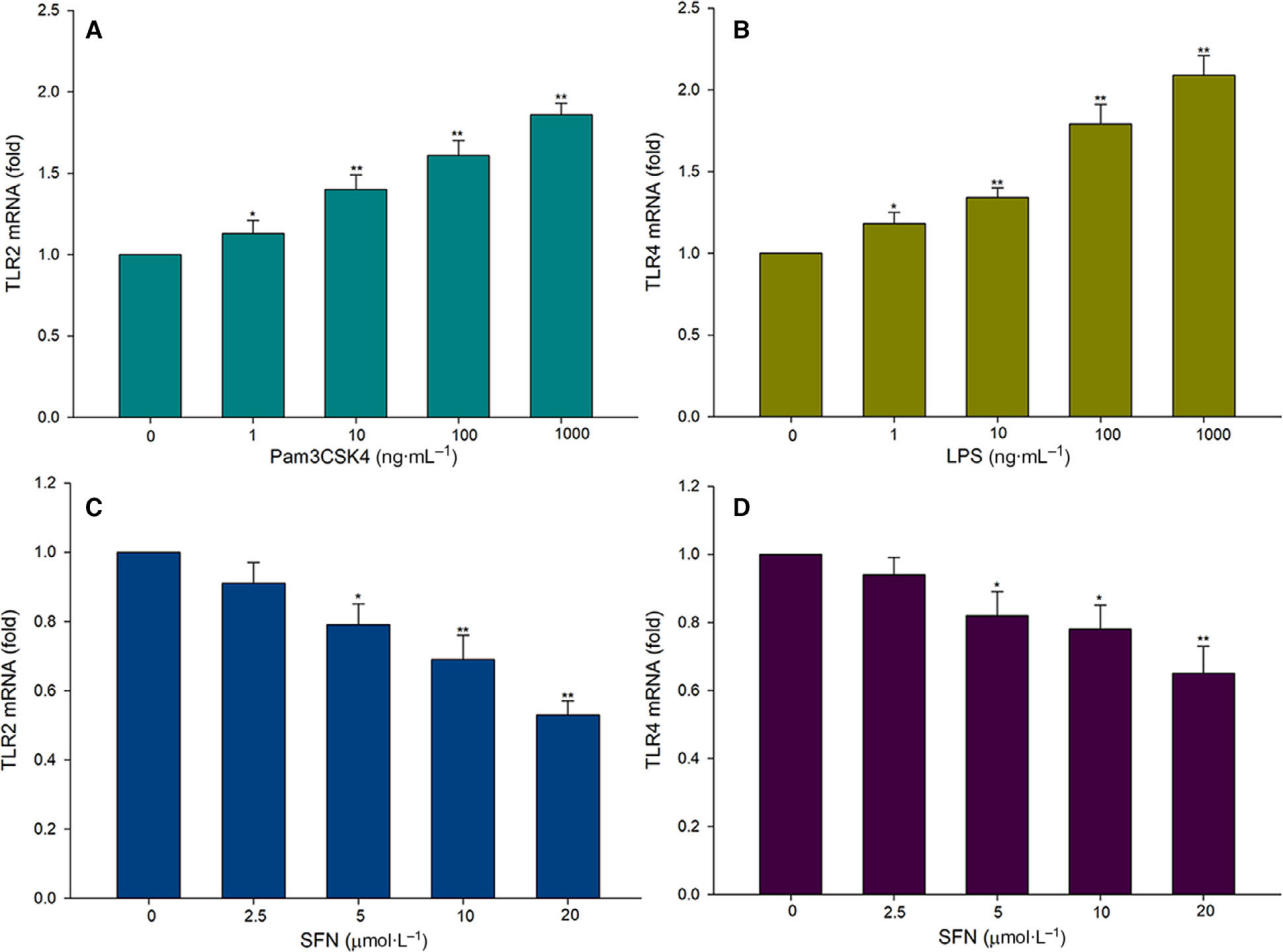
The expression levels of TLR2 and TLR4 in the MDMs of patients with COPD. MDMs were treated with agonist stimulation at the indicated concentrations for 6 h. (A) The relative expression of TLR2 was determined by quantitative real‐time PCR in MDMs treated with 0, 1, 10, 100 and 1000 ng·mL^−1^ Pam3CSK4. (B) The relative expression of TLR4 was determined by quantitative real‐time PCR in MDMs treated with 0, 1, 10, 100 and 1000 ng·mL^−1^ LPS. (C) The relative expression of TLR2 was measured by quantitative real‐time PCR in MDMs treated with 0, 2.5, 5, 10 and 20 μmol·mL^−1^ SFN. (D) The relative expression of TLR4 was measured by quantitative real‐time PCR in MDMs treated with 0, 2.5, 5, 10 and 20 μmol·mL^−1^ SFN. Data are presented as means ± SD of five independent experiments. Differences were assessed by one‐way ANOVA test. **P < 0.01, *P < 0.05.

To investigate whether TLR agonists and SFN can influence the expression of TLR2 and TLR4 in MDMs from patients with COPD, we performed quantitative real‐time PCR on RNA extracts. We added increasing concentrations (1–1000 ng·mL^−1^) of Pam3CSK4 or LPS to the cells for 6 h. The expression levels of TLR2 and TLR4 mRNA increased in a dose‐dependent manner (Fig. 1). We therefore selected a higher concentration of TLR agonists (1000 ng·mL^−1^ Pam3CSK4, 1000 ng·mL^−1^ LPS) for MDM treatments in all subsequent experiments.

To determine the effects of SFN on the expression of TLRs, we treated MDMs with increasing concentrations of SFN for 6 h. As shown in Fig. 1, SFN down‐regulated the expression of TLR2 and TLR4 mRNA in a concentration‐dependent manner. Because 20 μm·L^−1^ of SFN exposure showed maximal TLR2 and TLR4 inhibition, we selected this dose for all subsequent experiments.

### SFN down‐regulated the Pam3CSK4‐ or LPS‐induced expression of TLR2 and TLR4 in MDMs

**Fig. 2:**
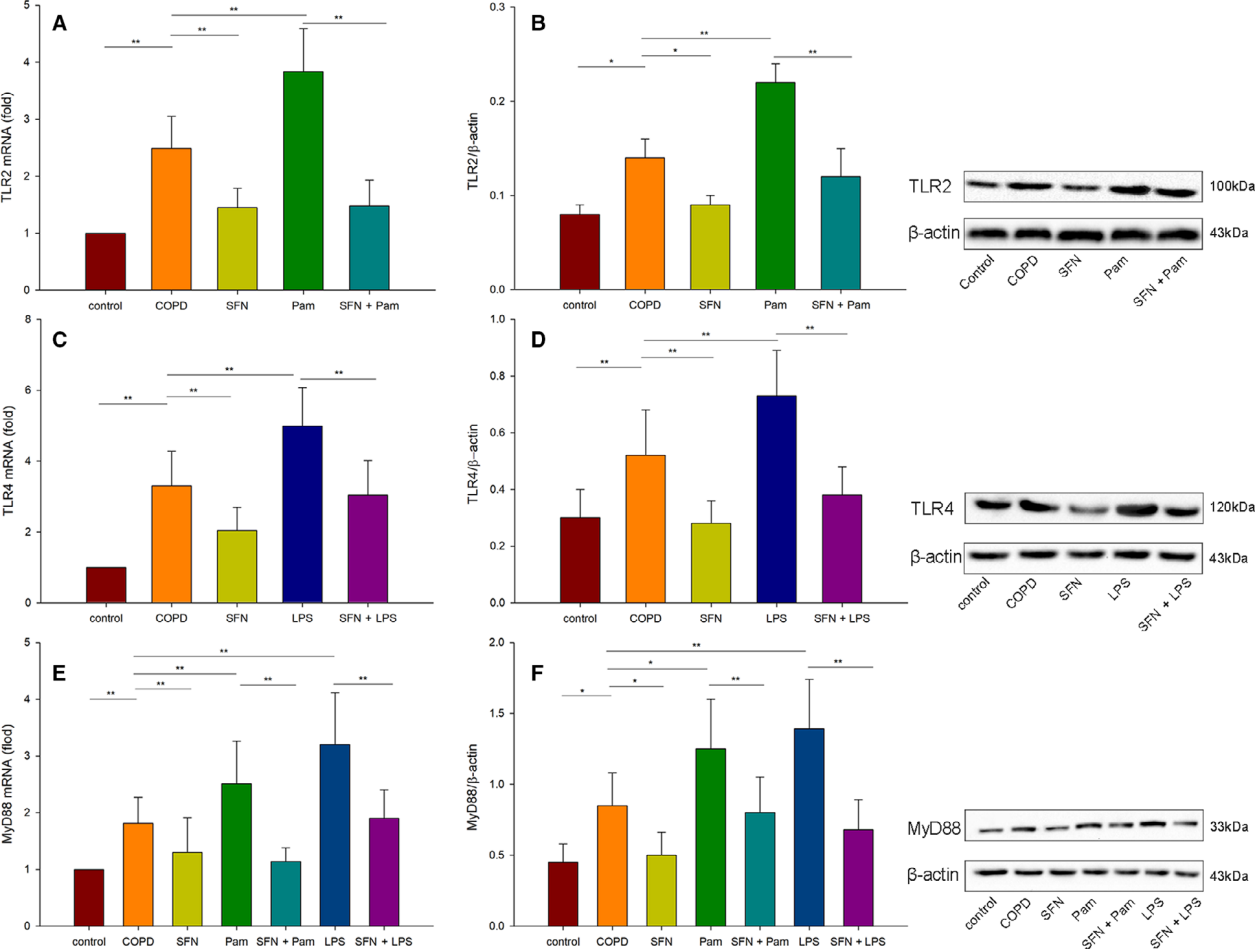
The inhibitory effect of SFN on TLR2, TLR4 and MyD88 mRNA and protein expression induced by Pam3CSK4 or LPS. Cells from the healthy control and COPD groups were cultured with RPMI 1640 medium. Cells in the other groups were stimulated with 1000 ng·mL^−1^ LPS, 1000 ng·mL^−1^ Pam3CSK4 (Pam), 20 μmol·L^−1^ SFN or cocultured with SFN and TLR agonists. Cells were cultured for 6 h for mRNA expression and for 24 h for protein expression. (A, B) The mRNA and protein levels of TLR2 were determined by quantitative real‐time PCR and western blot analysis. (C, D) The mRNA and protein levels of TLR4 were determined by quantitative real‐time PCR and western blot analysis. (E, F) The mRNA and protein levels of MyD88 were determined by quantitative real‐time PCR and western blot analysis. Data are presented as means ± SD. Differences were assessed by one‐way ANOVA test. **P < 0.01, *P < 0.05.

As shown in Fig. 2, patients with COPD had higher baseline expression levels of TLR2 and TLR4 compared with healthy control subjects. We examined the effect of SFN on Pam3CSK4‐ or LPS‐induced TLR2 and TLR4 expression by quantitative real‐time PCR for mRNA expression and by western blot analysis for protein expression. Results showed that both Pam3CSK4 and LPS increased the expression levels of TLR2 and TLR4 mRNA and protein compared with the COPD nontreatment group (*P* < 0.01). In contrast, SFN down‐regulated the mRNA and protein levels of TLR2 and TLR4. Pretreatment with SFN reduced the Pam3CSK4‐ or LPS‐induced expression of TLR2 and TLR4 (*P* < 0.05).

### SFN inhibited Pam3CSK4‐ or LPS‐induced MyD88 expression in MDMs

Next, we then examined whether SFN and TLRs agonists were able to influence the transcript expression of the TLRs signaling downstream adaptor (MyD88). As shown in Fig. 2, baseline MyD88 expression level was higher in patients with COPD than in healthy control subjects (*P* < 0.05). After incubation with Pam3CSK4 or LPS, respectively, we found that the mRNA and protein levels of MyD88 in MDMs from patients with COPD were up‐regulated (*P* < 0.05). Stimulation with SFN significantly reduced the expression of MyD88 in patients with COPD; SFN also inhibited the Pam3CSK4‐ or LPS‐induced expression of MyD88 (*P* < 0.01).

### SFN ameliorated Pam3CSK4‐ or LPS‐mediated cytokine production

**Fig. 3:**
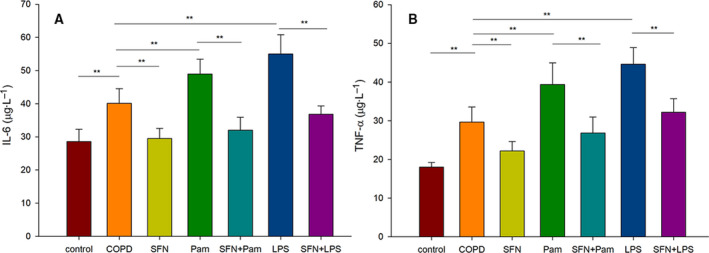
The effects of SFN on concentrations of inflammatory mediators in cell supernatants induced by Pam3CSK4 (Pam) or LPS. The levels of IL‐6 (A) and TNF‐α (B) in culture supernatants were measured by ELISA. These results are representative of 10 independent experiments. Data are presented as means ± SD. Differences were assessed by one‐way ANOVA test. **P < 0.01.

Finally, MDMs were challenged with Pam3CSK4 or LPS in the presence or absence of SFN for 24 h. Then we measured the concentrations of IL‐6 and TNF‐α in the cell supernatants. As shown in Fig. 3, the levels of IL‐6 and TNF‐α were higher in the COPD group than in the healthy group (*P* < 0.01). After Pam3CSK4 or LPS stimulation alone, there were tendencies for the higher secretion of IL‐6 and TNF‐α in patients with COPD. SFN suppressed the release of cytokines from MDMs in patients with COPD (*P* < 0.01). Simultaneously, SFN significantly reduced Pam3CSK4‐ or LPS‐induced IL‐6 and TNF‐α compared with Pam3CSK4 or LPS treatment groups (*P* < 0.01).

### Correlation analysis

The protein expression levels of TLR2, TLR4 and MyD88 were positively correlated with that of IL‐6 and TNF‐α in each group (*P* < 0.05 or *P* < 0.01) (Table 2).

**Table 2:** Analysis of the correlation between protein expression levels of TLR2, TLR4, MyD88, IL‐8 and TNF‐α in each group (*r* value). The correlation coefficients were obtained by the Pearson test.

	TLR2	TLR4	MyD88
IL‐6			
Control	0.780^a^	0.764^b^	0.790^a^
COPD	0.743^b^	0.680^b^	0.735^b^
SFN	0.768^b^	0.801^a^	0.735^b^
Pam3CSK4	0.783^a^		0.792^a^
SFN+Pam3CSK4	0.803^a^		0.815^a^
LPS		0.831^a^	0.801^a^
SFN+LPS		0.870^a^	0.815^a^
TNF‐α			
Control	0.759^b^	0.712^b^	0.755^b^
COPD	0.790^a^	0.801^a^	0.786^a^
SFN	0.801^a^	0.820^a^	0.805^a^
Pam3CSK4	0.820^a^		0.813^a^
SFN+Pam3CSK4	0.825^a^		0.863^a^
LPS		0.880^a^	0.820^a^
SFN+LPS		0.851^a^	0.830^a^

^a^ *P* < 0.01

^b^ *P* < 0.05.

## Discussion

COPD is a heterogeneous disease that is characterized by chronic inflammation and significant airflow obstruction that is incompletely reversible. AM plays an important role in chronic inflammation in patients with COPD. Activated AMs release more inflammatory mediators and chemokines. However, AMs are poorly available in humans to perform *in vitro* studies because of limited access to bronchoalveolar lavage. In addition, previous studies have shown the phenotype and functions of GM‐CSF‐induced MDMs closely resemble that of human AMs, indicating that GM‐CSF‐induced MDMs are useful to clarify the molecular mechanisms of human AMs [16, 17]. Therefore, MDM is often used as a cell model of AM in *in vitro* studies. This study also used an MDM model to investigate inflammatory mechanisms in patients with COPD.

Bacterial colonization of the airways in COPD is an important factor in disease progression, and some patients with COPD are colonized by bacteria during both the stable phase and exacerbations, which may lead to persistent inflammation in the airway [18]. It is generally known that the airways of patients with COPD are colonized by *S. pneumoniae*, *Haemophilus influenza*, *M. catarrhalis* and, in patients with severe COPD, *Pseudomonas aeruginosa* [19]. The bacterial PAMPs, LPS (a Gram‐negative bacterial cell wall component) and lipoprotein (a Gram‐positive bacterial cell wall component) are transduced through TLR4 and TLR2, respectively, thus promoting the activation of downstream MyD88 signaling pathways and increasing the secretion of proinflammatory mediators. In a previous study, Pons *et al*. [20] found that the expression of TLR‐2 was up‐regulated in peripheral blood monocytes of patients with COPD, either when clinically stable or during an exacerbation of the disease, as compared with people with normal lung function. Another study demonstrated that TLR4 expression was increased in the bronchial mucosa of patients with severe stable COPD compared with control subjects [21]. Consistent with these previous observations, we observed higher expression levels of TLR2 and TLR4 in the MDMs of patients with COPD compared with healthy subjects. In addition, the downstream expression of MyD88 and inflammatory cytokines in the MDMs of patients with COPD were also higher compared with healthy control subjects. These findings indicated that persistent inflammation may be related to the TLRs/MyD88 pathway activated by bacterial colonization.

Pam3CSK4, a synthetic bacterial lipoprotein, has been shown to induce TLR2 expression at both the mRNA and the protein levels and can also increase the expression levels of IL‐1β, IL‐6 and TNF‐α in monocytes [22]. Similarly, LPS, a main component of the cell wall of Gram‐negative bacteria, is usually recognized by TLR4 and induces proinflammatory reactions in numerous cell types. After recognizing specific ligands, TLR2 and TLR4 initiate intracellular signaling that is dependent on adaptor protein MyD88. In this study, we demonstrated that Pam3CSK4 or LPS up‐regulated the expression of TLR2 and TLR4, respectively, in MDMs from COPD, along with the increased expression of MyD88 and the subsequent increase of inflammatory mediators.

**Fig. 4:**
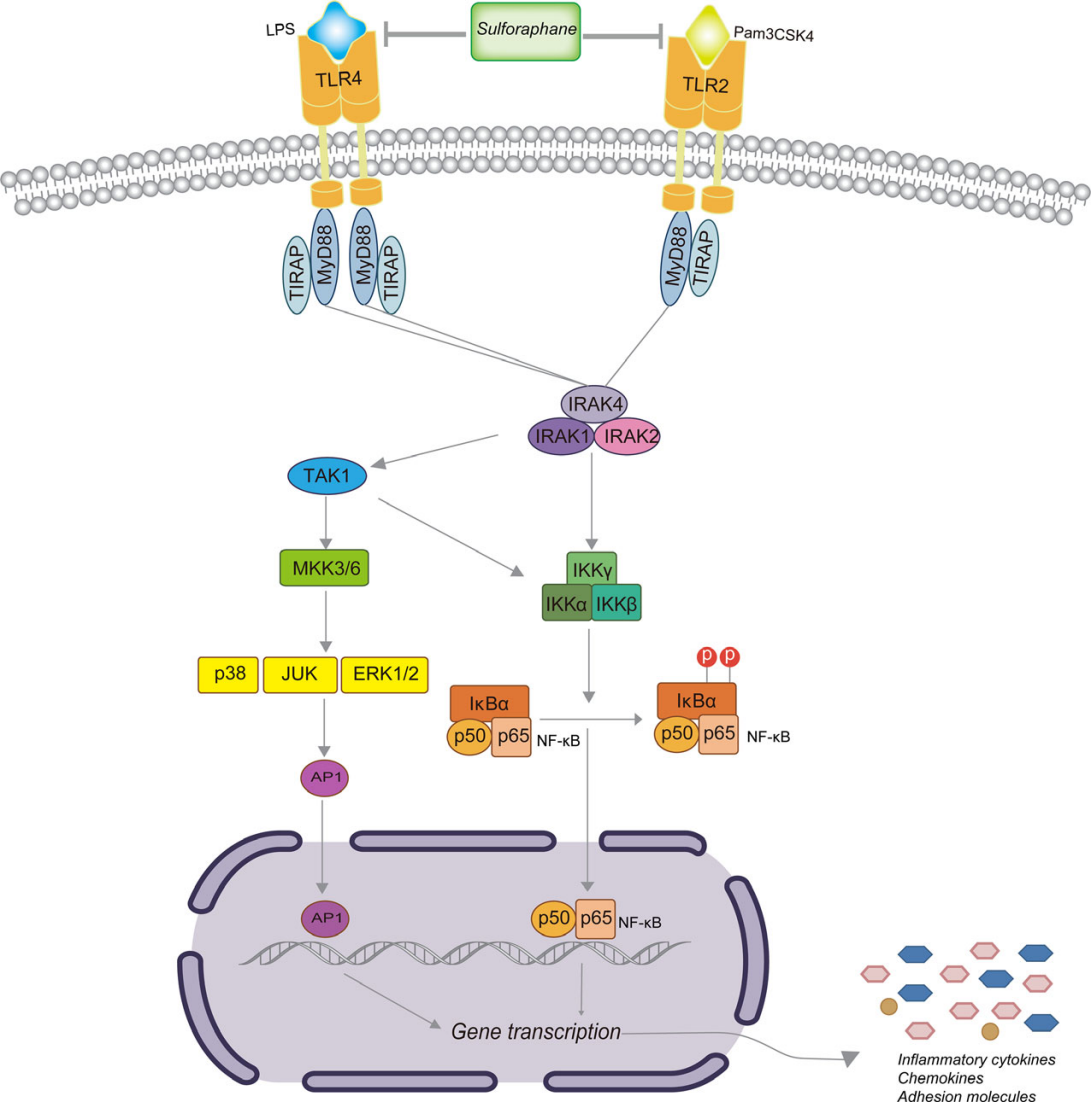
Sulforaphane inhibited inflammation via the TLR2, TLR4/MyD88 pathway in macrophage. AP1, activator protein 1; ERK 1/2, extracellular signal regulated kinase 1/2; JUK, c‐jun N‐terminal kinase; IKK, IκB kinase; IRAK, interleukin‐1 receptor‐associated kinases; TAK1, transforming growth factor beta‐activated kinase 1.

As a critical role in sensing PAMPs, reducing TLR2 and TLR4 can help to inhibit inflammation. Hoth *et al*. [23, 24] reported that TLR2 and TLR4 gene knockout mice both have reduced local and systemic inflammation in pulmonary contusion. Dasu *et al*. [22] showed that decreasing TLR2 and TLR4 expression inhibited the release of inflammatory factors in monocytes. In recent years, dietary phytochemicals have become the advantageous agents in the prevention and therapy of chronic diseases. We hypothesized that SFN could inhibit the inflammation of COPD in a TLR/MyD88 manner (Fig. 4). SFN, an aliphatic isothiocyanate derived primarily from broccoli, has emerged as a phytochemical with comparatively high bioavailability because of its low molecular weight [25]. SFN is a highly promising agent that has been studied extensively in cells and animals with regard to preventive and therapeutic effects. Previous research showed that SFN had multiple molecular targets to exert its anti‐inflammatory, antioxidant or anticancer activities [26]. In a previous study, Cho *et al*. [27] reported that SFN significantly reduced the magnitude of hyperoxia‐increased lung protein oxidation and lipid peroxidation in Nrf2^+/+^ mice, alleviating acute lung injury in an Nrf2‐dependent manner. Qin *et al*. [28] reported that SFN significantly blocked the phosphorylation of mitogen‐activated protein kinases and NF‐κB p65 and down‐regulated the expressions of proinflammatory mediators, including TNF‐α, IL‐1β, IL‐6, and inducible nitric oxide synthase in a mouse microglial cell line. Ruhee *et al*. [29] showed that SFN inhibited LPS‐induced inflammation in macrophages through the Nrf2/heme oxygenase‐1 signal pathway. Our results demonstrated that SFN suppressed the expression of TLR2 and TLR4 both at the mRNA and the protein levels, thus suggesting potential clinical applications against inflammation in COPD. Simultaneously, SFN exhibited more significant down‐regulation of MyD88 and the expression of the downstream cytokines, TNF‐α and IL‐6. Furthermore, SFN pretreatment on MDMs significantly reduced LPS‐ or Pam3CSK4‐induced expression of TLR2, TLR4, MyD88 and cytokines, thus conferring anti‐inflammatory activity in agonist‐stimulated macrophages.

In summary, SFN, a dietary isothiocyanate derived from cruciferous vegetables, displays a variety of beneficial bioactivities through multiple mechanisms. Our study has demonstrated that SFN inhibited the production of cytokines via the TLRs pathway in MDMs from patients with COPD. These targets manipulated by SFN may provide its extensive perspective in clinical application of COPD.

## Conflict of interest

The authors declare no conflict of interest.

## Author contributions

XZ and XL conceived and designed the project and performed the data analyses. XZ and HB contributed the materials and performed the experiments. XZ wrote the manuscript. XL contributed to the critical revision of the article. All authors approved the final version of the manuscript.

## Data Availability

The data that support the findings of this study are available from the corresponding author upon reasonable request.

## References

[1] Vogelmeier CF , Criner GJ , Martinez FJ , Anzueto A , Barnes PJ , Bourbeau J , Celli BR , Chen R , Decramer M , Fabbri LM *et al*. (2017) Global strategy for the diagnosis, management, and prevention of chronic obstructive lung disease 2017 report. GOLD executive summary. Am J Respir Crit Care Med 195, 557–582. 28128970 10.1164/rccm.201701-0218PP

[2] Rabe KF and Watz H (2017) Chronic obstructive pulmonary disease. Lancet 389, 1931–1940. 28513453 10.1016/S0140-6736(17)31222-9

[3] Polosukhin VV , Richmond BW , Du RH , Cates JM , Wu P , Nian H , Massion PP , Ware LB , Lee JW , Kononov AV *et al*. (2017) Secretory IgA deficiency in individual small airways is associated with persistent inflammation and remodeling. Am J Respir Crit Care Med 195, 1010–1021. 27911098 10.1164/rccm.201604-0759OC PMC5422646

[4] Angelis N , Porpodis K , Zarogoulidis P , Spyratos D , Kioumis I , Papaiwannou A , Pitsiou G , Tsakiridis K , Mpakas A , Arikas S *et al*. (2014) Airway inflammation in chronic obstructive pulmonary disease. J Thorac Dis 6 (Suppl 1), S167–S172. 24672691 10.3978/j.issn.2072-1439.2014.03.07 PMC3966160

[5] Kumar H , Kawai T and Akira S (2009) Toll‐like receptors and innate immunity. Biochem Biophys Res Commun 388, 621–625. 19686699 10.1016/j.bbrc.2009.08.062

[6] Du Q , Min S , Chen LY , Ma YD , Guo XL , Wang Z and Wang ZG (2012) Major stress hormones suppress the response of macrophages through down‐regulation of TLR2 and TLR4. J Surg Res 173, 354–361. 21109260 10.1016/j.jss.2010.10.016

[7] Kawai T , Adachi O , Ogawa T , Takeda K and Akira S (1999) Unresponsiveness of MyD88‐deficient mice to endotoxin. Immunity 11, 115–122. 10435584 10.1016/s1074-7613(00)80086-2

[8] Jacobs DM , Ochs‐Balcom HM , Zhao J , Murphy TF and Sethi S (2018) Lower airway bacterial colonization patterns and species‐specific interactions in chronic obstructive pulmonary disease. J Clin Microbiol 56, e00330‐18. 30045868 10.1128/JCM.00330-18 PMC6156310

[9] Cabello H , Torres A , Celis R , El‐Ebiary M , Puig de la Bellacasa J , Xaubet A , González J , Agustí C and Soler N (1997) Bacterial colonization of distal airways in healthy subjects and chronic lung disease: a bronchoscopic study. Eur Respir J 10, 1137–1144. 9163659 10.1183/09031936.97.10051137

[10] Jiang X , Liu Y , Ma L , Ji R , Qu Y , Xin Y and Lv G (2018) Chemopreventive activity of sulforaphane. Drug Des Devel Ther 12, 2905–2913. 10.2147/DDDT.S100534 PMC6141106 30254420

[11] Ruhee RT , Roberts LA , Ma S and Suzuki K (2020) Organosulfur compounds: a review of their anti‐inflammatory effects in human health. Front Nutr 7, 64. 32582751 10.3389/fnut.2020.00064 PMC7280442

[12] Harvey CJ , Thimmulappa RK , Sethi S , Kong X , Yarmus L , Brown RH , Feller‐Kopman D , Wise R and Biswal S (2011) Targeting Nrf2 signaling improves bacterial clearance by alveolar macrophages in patients with COPD and in a mouse model. Sci Transl Med 3, 78ra32. 10.1126/scitranslmed.3002042 PMC4927975 21490276

[13] Starrett W and Blake DJ (2011) Sulforaphane inhibits de novo synthesis of IL‐8 and MCP‐1 in human epithelial cells generated by cigarette smoke extract. J Immunotoxicol 8, 150–158. 21401388 10.3109/1547691X.2011.558529

[14] Zeng X , Liu X , Bao H , Zhang Y , Wang X , Shi K and Pang Q (2014) Effects of sulforaphane on Toll‐like receptor 4/myeloid differentiation factor 88 pathway of monocyte‐derived macrophages from patients with chronic obstructive pulmonary disease. Zhonghua Jie He He Hu Xi Za Zhi 37, 250–254. 24969711

[15] Taylor AE , Finney‐Hayward TK , Quint JK , Thomas CM , Tudhope SJ , Wedzicha JA , Barnes PJ and Donnelly LE (2010) Defective macrophage phagocytosis of bacteria in COPD. Eur Respir J 35, 1039–1047. 19897561 10.1183/09031936.00036709

[16] Akagawa KS , Komuro I , Kanazawa H , Yamazaki T , Mochida K and Kishi F (2006) Functional heterogeneity of colony‐stimulating factor‐induced human monocyte‐derived macrophages. Respirology 11 (Suppl), S32–S36. 16423268 10.1111/j.1440-1843.2006.00805.x

[17] Lescoat A , Ballerie A , Augagneur Y , Morzadec C , Vernhet L , Fardel O , Jégo P , Jouneau S and Lecureur V (2018) Distinct properties of human M‐CSF and GM‐CSF monocyte‐derived macrophages to simulate pathological lung conditions in vitro: application to systemic and inflammatory disorders with pulmonary involvement. Int J Mol Sci 19, 894. 10.3390/ijms19030894 PMC5877755 29562615

[18] Tufvesson E , Markstad H , Bozovic G , Ekberg M and Bjermer L (2017) Inflammation and chronic colonization of *Haemophilus influenzae* in sputum in COPD patients related to the degree of emphysema and bronchiectasis in high‐resolution computed tomography. Int J Chron Obstruct Pulmon Dis 12, 3211–3219. 29138549 10.2147/COPD.S137578 PMC5677300

[19] Dima E , Kyriakoudi A , Kaponi M , Vasileiadis I , Stamou P , Koutsoukou A , Koulouris NG and Rovina N (2019) The lung microbiome dynamics between stability and exacerbation in chronic obstructive pulmonary disease (COPD): current perspectives. Respir Med 157, 1–6. 31450162 10.1016/j.rmed.2019.08.012

[20] Pons J , Sauleda J , Regueiro V , Santos C , López M , Ferrer J , Agustí AG and Bengoechea JA (2006) Expression of Toll‐like receptor 2 is up‐regulated in monocytes from patients with chronic obstructive pulmonary disease. Respir Res 7, 64. 16606450 10.1186/1465-9921-7-64 PMC1458333

[21] Di Stefano A , Ricciardolo FLM , Caramori G , Adcock IM , Chung KF , Barnes PJ , Brun P , Leonardi A , Andò F , Vallese D *et al*. (2017) Bronchial inflammation and bacterial load in stable COPD is associated with TLR4 overexpression. Eur Respir J 49, 1602006. 28536249 10.1183/13993003.02006-2016

[22] Dasu MR , Riosvelasco AC and Jialal I (2009) Candesartan inhibits Toll‐like receptor expression and activity both in vitro and in vivo. Atherosclerosis 202, 76–83. 18495130 10.1016/j.atherosclerosis.2008.04.010 PMC2676176

[23] Hoth JJ , Wells JD , Brownlee NA , Hiltbold EM , Meredith JW , McCall CE and Yoza BK (2009) Toll‐like receptor 4‐dependent responses to lung injury in a murine model of pulmonary contusion. Shock 31, 376–381. 18665044 10.1097/SHK.0b013e3181862279 PMC2918369

[24] Hoth JJ , Hudson WP , Brownlee NA , Yoza BK , Hiltbold EM , Meredith JW and McCall CE (2007) Toll‐like receptor 2 participates in the response to lung injury in a murine model of pulmonary contusion. Shock 28, 447–452. 17558351 10.1097/shk.0b013e318048801a

[25] Fahey JW , Wade KL , Stephenson KK , Panjwani AA , Liu H , Cornblatt G , Cornblatt BS , Ownby SL , Fuchs E , Holtzclaw WD *et al*. (2019) Bioavailability of sulforaphane following ingestion of glucoraphanin‐rich broccoli sprout and seed extracts with active myrosinase: a pilot study of the effects of proton pump inhibitor administration. Nutrients 11, 1489. 10.3390/nu11071489 PMC6682992 31261930

[26] Houghton CA (2019) Sulforaphane: its “coming of age” as a clinically relevant nutraceutical in the prevention and treatment of chronic disease. Oxid Med Cell Longev 2019, 2716870. 31737167 10.1155/2019/2716870 PMC6815645

[27] Cho HY , Miller‐DeGraff L , Blankenship‐Paris T , Wang X , Bell DA , Lih F , Deterding L , Panduri V , Morgan DL , Yamamoto M *et al*. (2019) Sulforaphane enriched transcriptome of lung mitochondrial energy metabolism and provided pulmonary injury protection via Nrf2 in mice. Toxicol Appl Pharmacol 364, 29–44. 30529165 10.1016/j.taap.2018.12.004 PMC6658087

[28] Qin S , Yang C , Huang W , Du S , Mai H , Xiao J and Lü T (2018) Sulforaphane attenuates microglia‐mediated neuronal necroptosis through down‐regulation of MAPK/NF‐κB signaling pathways in LPS‐activated BV‐2 microglia. Pharmacol Res 133, 218–235. 29391237 10.1016/j.phrs.2018.01.014

[29] Ruhee RT , Ma S and Suzuki K (2019) Sulforaphane protects cells against lipopolysaccharide ‐stimulated inflammation in murine macrophages. Antioxidants (Basel) 8, 577. 10.3390/antiox8120577 PMC6943607 31766492

